# A case report of hypersomnia in tetrasomy X improved with medical therapy

**DOI:** 10.1002/ccr3.1440

**Published:** 2018-03-22

**Authors:** Dilip Jayaraman, Karen S. Carvalho, Daphne M Hasbani

**Affiliations:** ^1^ St. Christopher's Hospital for Children 160 East Erie Avenue Philadelphia Pennsylvania 19134; ^2^ Present address: Department of Neurology Einstein Medical Center 5401 Old York Road, Klein Building, Suite 404 Philadelphia Pennsylvania 19141

**Keywords:** All genetics, all sleep disorders, other hypersomnias, seizure, tetrasomy X

## Abstract

Tetrasomy X is a rare chromosomal anomaly in which sleep disorders have not been previously reported. We report on one patient with tetrasomy X and hypersomnia successfully treated with psychostimulant therapy. Sleep disorders are rarely reported in chromosomal anomalies. Clinicians should screen patients for sleep disorders and manage them appropriately.

## Introduction

Tetrasomy X is a rare chromosomal disorder manifesting with varying degrees of cognitive impairment, characteristic facial features, joint laxity, hypotonia, and other features. Sleep disorders have not been previously reported in patients with tetrasomy X. We report on one patient with tetrasomy X and hypersomnia successfully treated with psychostimulant therapy.

## Case Report

A 17‐year‐old female with a past medical history of tetrasomy X, ventricular septal defect, and intellectual disability was referred to our pediatric neurology clinic for evaluation of episodes of falling asleep. Over the previous 2 years, she had had 5–6 episodes of altered consciousness lasting for about 15 minutes during which she appeared to be falling asleep. She complained of extreme fatigue prior to some of the episodes. She was unable to talk during the episodes but could shake her head to indicate a response to questioning. Her parents had to help her move during these episodes but she neither had abnormal movements nor was she described as limp. There were no postictal symptoms. She also complained of excessive daytime sleepiness requiring a daily nap of 2–3 h in the afternoon despite sleeping 8–9 h per night. She denied snoring, nocturnal apneic events, cataplexy, sleep paralysis, or hypnagogic/hypnopompic hallucinations. Cardiac evaluation documented a small hemodynamically insignificant muscular ventricular septal defect without arrhythmia. Her paternal grandmother had seizures due to traumatic brain injury. Otherwise, there was no family history of seizures or sleep disorders.

On examination, she had characteristic dysmorphic features with an elongated face and a single palmar crease bilaterally. Her body mass index (BMI) was 26 kg/m^2^ (88th percentile). Neurological examination was unremarkable except for mild diffuse hypotonia. Cognitive assessment showed a full‐scale intelligence quotient of 69. Epworth Sleepiness Scale was 20. Epworth Sleepiness Scale is a tool to assess daytime sleepiness with scores ranging from 0 to 24. Scores 11–24 denote increasing levels of excessive daytime sleepiness.

Routine laboratory works ruled out other causes such as anemia and thyroid abnormalities. Magnetic resonance imaging (MRI) of the brain showed multiple nonenhancing subcortical white matter T2 signal hyperintensities of uncertain significance. Two routine electroencephalograms were normal. Polysomnography (PSG) was normal. Multiple sleep latency test (MSLT – <8 abnormal suggestive of hypersomnia) showed a mean sleep latency of 7.2 min with no sleep‐onset rapid eye movement (REM) sleep.

She was started on 10 mg extended‐release dextroamphetamine/amphetamine in the morning. This resulted in significant improvement in hypersomnolence in the morning but she still had fatigue and sleepiness in the late afternoon. Her morning dose of extended‐release dextroamphetamine/amphetamine was increased to 15 and 5 mg was added in the afternoon. Three months after being on this regimen, she reported resolution of sleepiness throughout the day and did not require naps. Additionally, her school performance improved with reported improvement in attention as well. No side effects were reported, though her BMI decreased to 22 kg/m^2^ (63rd percentile).

## Discussion

Tetrasomy X is an extremely rare chromosomal disorder that results from a nondisjunction event during gametogenesis or after conception. Girls with tetrasomy X have characteristic facial features, varying degrees of cognitive dysfunction, and skeletal and connective tissue disorders. The dysmorphic facial features include epicanthal folds, upslanting palpebral fissures, and hypertelorism. Abnormalities of teeth and enamel, radioulnar synostosis, and joint hyperflexibility characterize the connective tissue and skeletal disorders. Neurocognitive deficits, speech and language disorders, and executive dysfunction have been described 1.

Hypersomnia is the inability to stay awake and alert during the major waking episodes of the day, resulting in periods of irrepressible need for sleep or unintended lapses into drowsiness or sleep. Excessive daytime sleepiness has been well described in patients with other chromosomal abnormalities including Prader–Willi syndrome and Smith–Magenis syndrome 2, 3. Hypersomnia has not previously been reported in patients with tetrasomy X.

Patients with hypersomnolence, including those with narcolepsy, idiopathic hypersomnia, and sleep‐disordered breathing, are often treated with psychostimulants to good effect. Modafinil and armodafinil are approved by the Food and Drug Administration (FDA) for the treatment of hypersomnia associated with narcolepsy in adults. These medications block reuptake of dopamine in the nerve terminus by binding to the dopamine transporter. Amphetamine is also FDA‐approved for the treatment of hypersomnia associated with narcolepsy in adults. Dextroamphetamine/amphetamine carries this indication as well but is also FDA‐approved for the treatment of children with narcolepsy. This medication blocks reuptake of dopamine and norepinephrine in the nerve terminal. Other stimulants which carry indications for treatment of attention‐deficit hyperactivity disorder (ADHD), including methamphetamine, methylphenidate, and dexmethylphenidate, are not FDA‐approved for the treatment of hypersomnia associated with narcolepsy in adults or children. Most of these stimulants are not indicated for the treatment of hypersomnia due to causes other than narcolepsy, but the American Academy of Sleep Medicine has recommended these medications as options for the treatment of hypersomnia 4. Indeed, there is some evidence that psychostimulants can ameliorate hypersomnia in certain chromosomal disorders 5.

Our patient had hypersomnia by clinical findings, which was confirmed by MSLT. Other causes of hypersomnia such as sleep‐disordered breathing and insufficient sleep syndrome were ruled out by history and PSG. Narcolepsy was ruled out by history and MSLT. There was no recent contributing history of infections or head trauma. There was no history of substance abuse or sedating medications, and she had no other systemic illnesses that could have led to hypersomnolence.

The pathogenesis of hypersomnia in polysomy X is unknown as it has not been reported previously. Low levels of hypocretin, produced in the hypothalamus, have been reported in other conditions associated with hypersomnia including narcolepsy, Kleine–Levin syndrome, and Prader–Willi syndrome 6, 7. Growth and hormonal abnormalities in patients with tetrasomy X suggest potential hypothalamic involvement, but this is not yet well understood likely due to the rarity of the condition 8. Indeed, dysfunction of the hypothalamic‐gonadal axis has been well described in the more common trisomy X with the suggestion of a central cause 9. There is a paucity of reports on structural brain abnormalities in tetrasomy X, but one report demonstrates nonspecific multifocal areas of T2 signal hyperintensity in the white matter similar to what was seen in our patient 10. There are no reports of hypothalamic or brainstem abnormalities noted on imaging studies in either of these conditions.

Despite the unclear etiology of hypersomnia in our patient with tetrasomy X, we herein demonstrate that alertness and quality of life in this condition can be improved with CNS stimulants. In our patient, school performance was greatly improved as well. Other patients with genetic disorders and hypersomnia may also benefit from off‐label use of CNS stimulants. More research is needed to identify the pathogenesis of sleep disorders in patients with tetrasomy X and other chromosomal abnormalities. Clinicians should screen patients with tetrasomy X for sleep disorders and manage them appropriately.

## Conflict of Interest

None declared.

## Authorship

DJ: drafted the manuscript, contributed to the conception, design, conduct, analysis, and interpretation of this work.

DMH: contributed to the conception, design, conduct, analysis, and interpretation of this work. Reviewed and approved this manuscript.

KSC: contributed to the conception, design, conduct, analysis, and interpretation of this work. Reviewed and approved this manuscript.
